# A machine learning framework for classifying lipids in untargeted metabolomics using mass-to-charge ratios and retention times

**DOI:** 10.1007/s11306-025-02343-y

**Published:** 2025-10-18

**Authors:** Christelle Colin-Leitzinger, Yonatan Ayalew Mekonnen, Isis Narvaez-Bandera, Vanessa Y. Rubio, Dalia Ercan, Eric A. Welsh, Lancia N. F. Darville, Min Liu, Hayley D. Ackerman, Julian Avila-Pacheco, Clary B. Clish, Kevin Hicks, John M. Koomen, Nancy Gillis, Brooke L. Fridley, Elsa R. Flores, Oana A. Zeleznik, Paul A. Stewart

**Affiliations:** 1 Department of Cancer Epidemiology, Moffitt Cancer Center, Tampa, FL USA; 2 Department of Molecular Oncology, Moffitt Cancer Center, Tampa, FL USA; 3 Department of Biostatistics and Bioinformatics, Moffitt Cancer Center, Tampa, FL USA; 4 Biostatistics and Bioinformatics Shared Resource, Moffitt Cancer Center, Tampa, FL USA; 5 Proteomics and Metabolomics Core, Moffitt Cancer Center, Tampa, FL USA; 6 Broad Institute of MIT and Harvard, Cambridge, MA USA; 7 Department of Nutrition and Integrative Physiology, University of Utah, Salt Lake City, UT USA; 8 Division of Health Services and Outcome Research, Children’s Mercy, Kansas City, USA; 9 Channing Division of Network Medicine, Brigham and Women’s Hospital and Harvard Medical School, Boston, MA USA; 10 Huntsman Cancer Institute, University of Utah, Salt Lake City, UT USA

**Keywords:** LC–MS, Machine learning, Mass–to–charge ratio, Retention time, Unknown metabolites

## Abstract

**Introduction:**

The identification of unknown metabolites remains a major challenge in untargeted metabolomics using liquid chromatography-mass spectrometry (LC–MS). This process typically depends on comparing mass spectral or chromatographic data to reference databases or deciphering complex fragmentation in tandem mass spectra. While current machine learning methods can predict metabolite structures using MS/MS (MS2) data, no approaches, to our knowledge, use only mass-to-charge ratio (m/z) and retention time (RT) from LC–MS data.

**Objective:**

To explore the potential of using the mass-to-charge ratio (m/z) and retention time (RT) from LC–MS data as standalone predictors for metabolite classification and propose a modeling framework which can be implemented internally on standalone datasets.

**Methods:**

We trained machine learning models on 20 mouse lung adenocarcinoma tumor samples with 7,353 features and validated them on a dataset of 81 samples with 22,000 features. A total of 120 combination of preprocessors and models were assessed. Features were classified as “lipid” or “non-lipid” based on the Human Metabolome Database (HMDB) taxonomy, and model performance was assessed using accuracy, area under the receiver operating characteristic curve (AUC), and area under the precision-recall curve (PR). We replicate the process in an independent dataset generated using human plasma samples.

**Results:**

We classified untargeted LC–MS features as “lipid” or “non-lipid” per the HMDB super class taxonomy and evaluated model performance. A framework including steps to choose the preprocessors and models for metabolite classification was designed. In our lab, tree-based models demonstrated superior performance across all metrics, achieving high accuracy, AUC, and PR which was consistent with the independent dataset.

**Conclusion:**

Our results demonstrate that metabolites can be classified as “lipid”, “non-lipid” using only m/z and RT from untargeted LC–MS data, without requiring MS2 spectra. Although this study focused on lipid classification, the approach shows potential for broader application, which warrants further investigation across diverse compound classes, detection methods, and chromatographic conditions.

**Supplementary Information:**

The online version contains supplementary material available at 10.1007/s11306-025-02343-y.

## Introduction

Advances in liquid chromatography and mass spectrometry now enable precise quantification of metabolites. Both targeted and untargeted methods can be used to identify and quantify metabolites (Kaczmarek et al., 2023). Unknown metabolites are found in every untargeted metabolomics experiment, yet their identification remains a significant challenge. Unknown metabolites, also called unidentified metabolites, are small molecules that can be detected, but whose chemical identity is not known. By some estimates, 90% of the features detected by liquid chromatography-mass spectrometry (LC–MS) metabolomics are unknown signals (Bowen et al., 2011). Identification of these unknowns relies on the comparison of spectral or chromatographic properties to reference databases or deciphering complicated fragmentation spectra. However, a considerable portion of unknown ion signals cannot be identified due to the diverse chemical structures, multiple adducts (*e.g.*, protonated, sodiated, dimers, etc.), artifactual chemical modifications (*e.g.*, gas phase water loss), background from environmental contaminants, and limited annotations of the metabolome. Combinatorial complexity further complicates this problem, as a given *m/z* value of an unknown metabolite can correspond to tens, if not hundreds, of possible metabolites. As a result, unknown metabolites significantly hinder the interpretation and understanding of metabolomics data since they cannot be directly linked to known biological functions.

Machine learning is increasingly used to make predictions about the properties of metabolites (Cripan et al., 2024; Galal et al., 2022). Given a chemical structure, machine learning approaches can predict the retention times (RT), fragmentation patterns, pathways, or interacting partners of a metabolite. Tools like CSI:FingerID (Duhrkop et al., 2015) and SIRIUS (Duhrkop et al., 2019) use machine learning to identify unknown metabolites by predicting their structure, but they require MS/MS (MS2) data as input. To our knowledge, no existing machine learning methods identify unknown metabolites from LC–MS data using only *m/z* and RT. Existing tools such as Mummichog (Li et al., 2013), PIUMet (Pirhaji et al., 2016), and BAUM (Ma et al., 2024) leverage network-based approaches to functionally interpret MS1-level metabolomics data by mapping significant features to metabolic pathways. In contrast, our approach directly utilizes only *m/z* and retention time to identify unknown metabolites without relying on prior pathway annotations. Since and RT are intrinsically linked to the chemical and physical characteristics of metabolites, we hypothesized that these features alone could be predictive of metabolite class (Rivas-Ubach et al., 2018). To test this hypothesis, we labeled identified features from untargeted LC–MS metabolomics of mouse tumors as 'lipid' and 'non-lipid'. Lipids are a class of hydrophobic, non-polar molecules, including fatty acids and steroids, that are chemically and physically distinct from polar metabolites. These differences result in different RT profiles providing an ideal test case for evaluating our hypothesis.

We leveraged an untargeted LC–MS metabolomics dataset comprising metabolite profiles from tumor samples, encompassing both discovery and validation sets, to investigate the discriminatory power of various machine-learning models. We evaluated the performance of ten machine learning models, including decision trees, support vector machines, random forests, and logistic regression, to maximize predictive performance. Additionally, we assess the impact of different data preprocessing techniques on model performance, such as data reduction, normalization, scaling, and balancing. These findings lay the foundation for future work using minimal data (*e.g.*, only *m/z* and RT) to predict the classes of unknown metabolites without requiring MS2. This predictive capability significantly streamlines the metabolite identification process by narrowing down the search space when querying an *m/z* against reference databases like the Human Metabolome Database (HMDB), thereby enhancing the efficiency and accuracy of metabolomics studies.

## Methods

### Reagents and chemicals

All solvents and chemicals are LC–MS grade unless otherwise noted. Ammonium hydroxide and ammonium carbonate were obtained from Sigma Aldrich. Water, methanol (MeOH), and acetonitrile (ACN) were obtained from VWR. The Metabolomics Quality Control (QC) kit, which contains 14 stable isotope-labeled metabolite standards, was obtained from Cambridge Isotope Labs and used as an internal standard (IS); it includes the following labeled compounds: L-Alanine (^13^C_3_, 99%), L-Leucine (^13^C_6_, 99%), L-Phenylalanine (^13^C_6_, 99%), L-Tryptophan (^13^C_11_, 99%), L-Tyrosine (^13^C_6_, 99%), Caffeine (^13^C_3_, 99%), D-Glucose (^13^C_6_, 99%), Benzoate (^13^C_6_, 99%), Citrate (^13^C_3_, 99%), Octanoate (^13^C_8_, 99%), Propionate (^13^C_3_, 99%), Stearic acid (^13^C_18_, 98%), Succinic acid (^13^C_4_, 99%) and D-Sucrose (^13^C_6_, 98%).

### Sample preparation

Frozen tissue samples were pulverized and maintained on ice for the duration of the liquid–liquid extraction. Pulverized tissues had 2 µL of the Metabolomics QC kit standards added immediately followed by protein precipitation using 500 µL of ice-cold aqueous 80% MeOH, previously chilled for 1 h at -80° C. Samples were vortexed thoroughly and incubated at -20° C for 30 min before centrifugation at 18,800 × g (Microfuge 22R, Beckman Coulter) at 4° C for 10 min. After centrifugation, the supernatant was transferred to a new microcentrifuge tube and dried by vacuum concentration at room temperature. The precipitated protein pellets were resolubilized using aqueous 20 mM HEPES with 8 M urea for protein quantification with a Bradford assay. Dried metabolite extract samples were reconstituted using aqueous 80% MeOH using volumes normalized to protein concentrations. Reconstituted samples were vortexed and incubated at -20° C for 10 min before being transferred to a glass LC vial for analysis. The independent dataset was generated using samples from human plasma (Zeleznik et al., 2020). Metabolites were profiled at the Broad Institute of MIT and Harvard (Cambridge, MA) using liquid chromatography tandem mass spectrometry (LC–MS/MS) as described previously (Mascanfroni et al., 2015; O'Sullivan et al., 2017; Paynter et al., 2018; Townsend et al., 2013).

### UHPLC-HRMS metabolomics

Ultra-high performance liquid chromatography (UHPLC) was done using a Thermo Scientific Vanquish interfaced with a Q Exactive HF high-resolution mass spectrometer (Thermo Scientific, San Jose, CA). Chromatographic separation was done either on a SeQuant ZIC-pHILIC column (4.6 mm ID × 150 mm length, 5 µm particle size) protected by a SeQuant ZIC-pHILIC guard column (4.6 mm ID × 20 mm length, 5 µm particle size) (Millipore Sigma, Burlington, MA) or Atlantis Premier BEH Z-HILIC VanGuard FIT column (2.1 mm ID × 150 mm length, 2.5 µm particle size) (Waters, Milford, MA). Gradient conditions were adapted from previously published methods (Almutairi et al., 2024; Pluskal et al., 2010). Briefly, mobile phase A was aqueous 10 mM ammonium carbonate with 0.05% ammonium hydroxide, and mobile phase B was acetonitrile. The run began at 80% B, with a linear increase over 13 min to reach 20% B. It then held isocratically at 20% B for 2 min, before returning to the original conditions within 0.1 min, where it equilibrated for an additional 4.9 min, bringing the total run time to 20 min. The column was maintained at a temperature of 30° C with a flow rate of 250 µL/min. All samples were acquired in positive and negative ionization mode with a 2 µL injection volume for each analysis. Mass spectrometry was performed using heated electrospray ionization (HESI) operating at a spray voltage of 3.5 kV and 3.0 kV for positive and negative mode, a capillary temperature of 325° C, sheath gas and auxiliary gas of 50 and 10 arbitrary units, respectively. The mass range for acquisition was 65 – 900 m*/z* in positive and negative ionization mode with a mass resolution of 120,000.

To demonstrate the adaptability of our approach, we analyzed an independent, published plasma metabolomics dataset (Zeleznik et al., 2020) generated using hydrophilic interaction liquid chromatography coupled with high-resolution mass spectrometry (HILIC-HRMS). Metabolites were extracted with isotope-labeled internal standards and analyzed in positive ionization mode with gradient separation. Comprehensive methodological details are available in the original publications (Mascanfroni et al., 2015; O'Sullivan et al., 2017; Paynter et al., 2018; Townsend et al., 2013).

### Data processing

Data were converted to open-source mzXML files using Raw Converter (Scripps) and then imported into MZmine 3.53 for feature finding and alignment. Mass detection was set to a 10 ppm window with a 0.25 min RT tolerance. Metabolite identification was performed using an in-house RT standard library. Aligned and annotated data were exported using peak height values. For each positive and negative ion mode dataset, rows with fewer than 2 high-quality pre-gap-filled features were removed as low quality. Each dataset was then normalized separately with iterative rank order normalization, IRON (Welsh et al., 2013), excluding heavy-labeled and unidentified rows from median sample identification (findmedian –pearson –iron-exclusions = unidentified_row_ids.txt –iron-spikeins = heavy_row_ids.txt) and normalization training (iron_generic –proteomics –iron-exclusions = unidentified_row_ids.txt –iron-spikeins = heavy_row_ids.txt). Normalized positive and negative ion mode datasets were then merged together and log2 transformed, treating zero abundances as missing data. Each row was then annotated, through fuzzy compound name matching, against our internal manually curated HMDB, KEGG, and PubChem identifier table.

### Data characteristics and cleaning

Data elements critical for this study include RT and *m/z* ratio measured by LC–MS, and HMBD IDs of the identified metabolites. To train our machine learning algorithm, we used a discovery dataset including data on 20 mouse lung adenocarcinoma tumor samples for which 7,353 features were detected. For validation, we use a dataset that includes 81 mouse tumor samples and 22,000 total features which had more than 45% of unique identified features in common with the discovery dataset (*e.g.* 188 unique HMDB IDs present in both dataset; 221 unique HMDB IDs in the discovery dataset and 412 unique HMDB IDs present in the validation dataset). After the data cleaning described below, 294 and 582 metabolites were identified for the discovery and validation datasets, respectively which shared 183 annotations (Table S1). To address RT across studies and instrumentation, we converted RT values into corresponding solvent composition percentages. This approach provides a more consistent and reproducible metric across different experimental setups. For improved reproducibility across studies, more specifically the variation of RT between study and apparatus, we calculated the solvent composition to substitute RT following the formula:

For solvent A (aqueous):

Given: At time* t* = 0, *y* (percentage) = 20% A and, at time* t* = 13, *y* (percentage) = 80% A.

Calculating the slope gives 4.615, since at *t* = 0, *y* = 20%, then *b* = 20, then accounting the delay causedby the system’s dead volume (${t}_{delay}$);

$$A\left(t\right)=4.615\left(t-{t}_{delay}\right)+20 for {t}_{delay} \le t\le 13+ {t}_{delay}$$

For solvent B (organic):

$$B\left(t\right)=100-\left(4.615 \left(t-{t}_{delay}\right)+20\right) for {t}_{delay}\le t \le 13+ {t}_{delay}$$

Data were linked to HMDB v5, downloaded from Wishart et al. (2022). Data elements extracted for our study include the HMDB IDs and taxonomy super class classification. The latter was used to classify identified metabolites as lipids ("lipid" *vs* "non-lipid") which will be used as the outcome of the algorithms.

Metabolites with a RT ≤ 1 min or ≥ 14 min were excluded to mitigate false detection during set up, washing, and equilibrating the HPLC column. To minimize overlap in identified metabolites between both polarities, preference was given to include the negatively charged identified metabolites if detected in both polarities (positive and negative) due to the larger number of identifications found in the negative ion mode dataset.

### Machine learning prediction

The discovery dataset, consisting solely of identified metabolites, was split into training and testing datasets using a proportion of 75/25% with a stratified sampling for the outcome variable. A tenfold cross-validation with the same stratified sampling was used on the training data to conduct the initial modeling. We used a combination of 10 models (naïve Bayes, decision tree and trimmed decision tree, support-vector machine, random forest, boosted tree, lasso regression, ridge regression, elastic net, and nearest neighbor) and together with 12 different data preprocessing approaches to train and test using the tidymodels package in R (version 1.1.1, R 4.3.2) (Kuhn et al., 2020). The data was used in its original format and with preprocessing modifications. The preprocessing methods included reducing the data by removing correlated variables and standardizing it through normalization or scaling. Additionally, we used an augmentation preprocessor using a nearest neighbor algorithm to create new synthetic observations similar to the data to balance the outcome variable. These preprocessors were tested individually and in combination – for example, data reduction with data augmentation, or data reduction combined with both data augmentation and data normalization. The hyperparameters for each model were tuned using the default grid search and selected to maximize the accuracy across the grid. The best preprocessor was selected based on the accuracy performance of each model in tenfold cross-validation on the training set.

We then applied the 10 models to the testing dataset and evaluate their performance based on accuracy, ROC AUC, and PR AUC for classifying lipid/non-lipid categories. Finally, the models were ranked based on these metrics, and the 4 best models, based on the rank sum, were applied to the identified metabolites in the validation dataset. A step-by-step summary of the workflow is provided in Figure S1. The code for the machine learning classification framework is available at: https://github.com/biodatalab/leitzinger_2025.git. To further demonstrate the versatility of our approach, we also included an independent dataset derived from human plasma samples of healthy women, generated under distinct experimental conditions (*e.g.*, positive ionization mode, 3 µm Atlantis HILIC column) (Zeleznik et al., 2020).

## Results and discussion

### Metabolites data description and cleaning

**Fig. 1:**
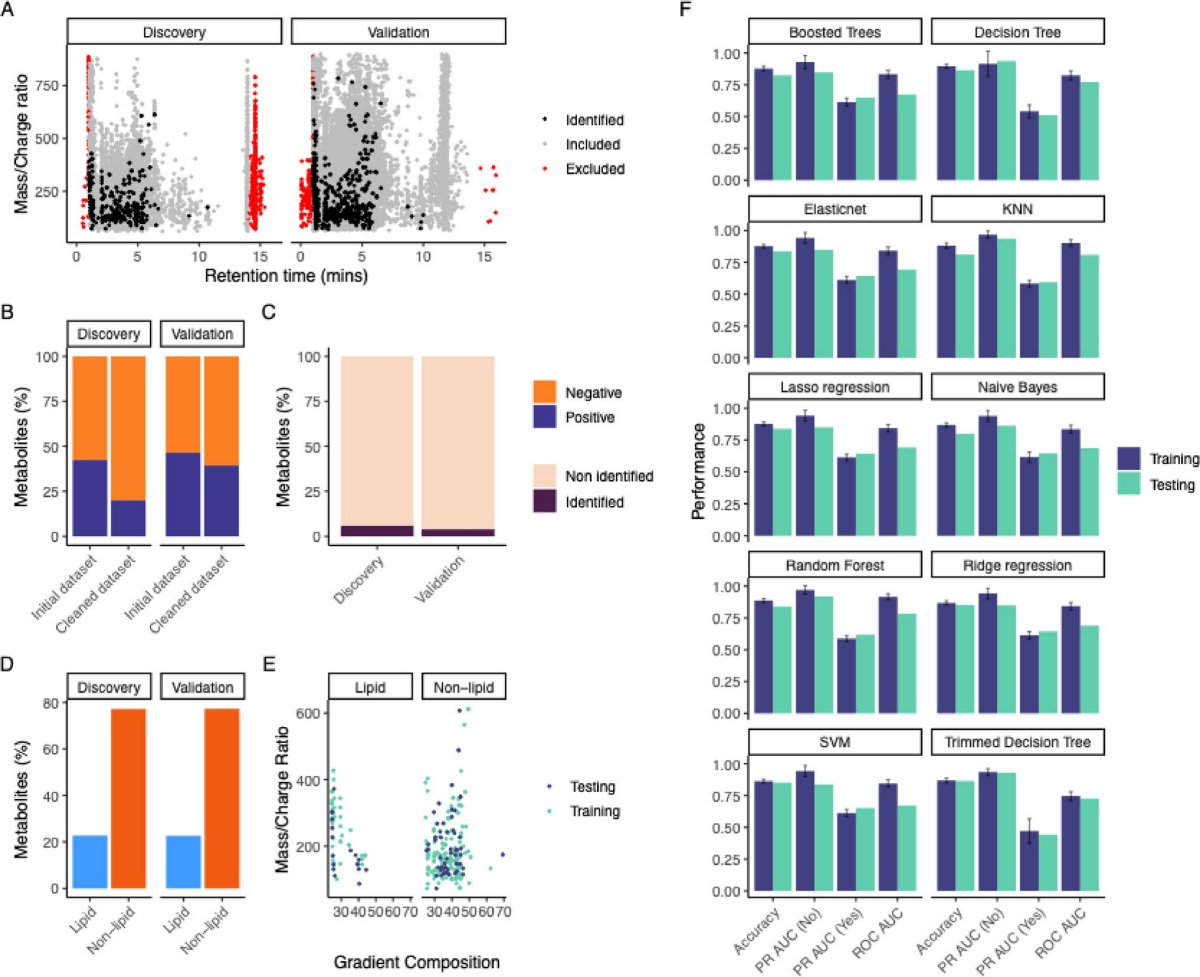
Data description. **A**. Scatter plot representing mass/charge ratio and retention time in the discovery and validation data before data cleaning. In red, metabolites excluded during the cleaning and in grey are the clean data points that can be included in the modeling. Metabolites identified in HMDB used in the modeling are depicted in black. **B**. Bar plot representing the percentage of metabolites charged positively and negatively before and after cleaning. **C**. Bar plot representing the proportion of identified vs non-identified metabolites in discovery and validation datasets. **D**. Bar plot representing the proportion of “non-lipid” and “lipid” metabolites in discovery and validation datasets. **E**. Scatter plot representing mass/charge ratio and gradient composition distribution for each outcome “non-lipid” and “lipid”. Visualization of the data points split between training and testing datasets is depicted in green and blue, respectively. **F**. Performance of 10 models on the training and testing set using accuracy, ROC AUC, PR AUC (“non-lipid”), PR AUC (“lipid”). Models, including naïve Bayes, decision tree, trimmed decision tree, support-vector machine (SVM), random forest, boosted tree, logistic regression (lasso, ridge), elastic net, and nearest neighbor (KNN), were evaluated on the 4 metrics accuracy, ROC AUC, PR AUC (“non-lipid”), PR AUC (“lipid”)

The discovery dataset includes 7,353 features detected while the validation dataset includes 22,000 detected features. To mitigate false detection during HPLC apparatus setup, washing, and equilibrating, metabolites with an RT ≤ 1 min, or ≥ 14 min were excluded (Fig. 1A). A greater proportion of the total features in both datasets were obtained from the negative mode data, with 4,240 features in the discovery dataset and 11,796 in the validation dataset. For metabolites with annotations that were detected in both polarities, positively charged metabolites were chosen for exclusion, which subsequently increased the percentage of negatively charged metabolites contributing to the datasets from 58 to 80% in the discovery data and from 54 to 61% in the validation data (Fig. 1B, C).

After cleaning, 4,887 and 14,611 metabolites were detected in the discovery and validation datasets, respectively. Identification using an in-house standard library of over 900 metabolites based on RT and exact mass-to-charge ratios was able to assign 6% and 4% of the total datasets with 294 and 582 identified metabolites in the discovery and validation datasets (Fig. 1B). All identifications between the two datasets were distributed within the 70 – 800 m/z range. In both acquired datasets, the total identifications were primarily comprised of organic acids and related derivative molecules (37% and 31%, respectively) with the second largest class comprised of lipids or other lipid-like molecules, such as those containing hydrophilic head groups and hydrophobic tails, with approximately 23% of the identifications in this class in each dataset (Fig. 1D and Table S2).

### Performance evaluation for each model and preprocessor

In our initial modeling, we compared the performance of 10 machine learning models and four data preprocessing techniques on a tenfold cross-validation of the training dataset. The models included naïve Bayes, decision tree, trimmed decision tree, support-vector machine (SVM), random forest, boosted tree, logistic regression (lasso, ridge), elastic net, and nearest neighbor (KNN). The 4 preprocessing techniques involved reducing (*i.e.*, removing correlated variables), normalizing or scaling the data, and balancing the data individually or in combination. We also used the model without applying any preprocessors resulting in 12 different preprocessing conditions. Ultimately, 120 model-preprocessor combinations were evaluated.

Accuracy, ROC AUC and PR AUC (for lipids and non-lipids) were used to select the best preprocessors for each model. For all 10 models, the metrics shows comparable performances across preprocessors including the models built using the raw data without preprocessing (referred to hereafter as "basic preprocessing"). No performance improvement was observed when applying preprocessing steps such as reduction, normalization, scaling, or their combinations (Figure S2A-D). Balancing the data, generating synthetic samples for the minority class (*i.e.*,“lipid”) to addresses the class imbalance, always slightly decreased the accuracy metric and its combination with other preprocessing techniques decreased it even further (Figure S2A-D).Given these results, and to preserve the original structure of the data, we proceeded with basic preprocessing for downstream analyses.

The hyperparameters selected for achieving optimal accuracy varied for each model using the basic preprocessing and are described in Table S3. Based on these findings, we pursued the evaluation of the models with minimal changes to the data, without preprocessing, and we again applied the 10-machine learning models on the training set and evaluated them on the testing set. Data characteristics between training and testing sets were similar (Fig. 1E and Table S2). We ranked these models based on accuracy, area under the Receiver Operating Characteristic Curve (ROC AUC), and area under the Precision Recall curve (PR AUC) performance for each outcome (“lipid” and “non-lipid”) (Table 1). Decision tree models (accuracy = 0.865 for trimmed and un-trimmed models) consistently outperformed other models in accuracy, followed by ridge regression (0.851), SVM (0.851), random forest (0.838), elastic net (0.838), lasso regression (0.838), boosted tree (0.824), nearest neighbor (0.811), and naïve bayes (0.797) (Fig. 1E and Table 1). The performance for ROC AUC ranked nearest neighbor (0.809) the highest followed by random forest (0.782), decision tree (0.771), and trimmed decision tree (0.727) (Fig. 1E, Figure S3, and Table 1). ROC AUC is commonly used to evaluate models, but in the case of unbalanced data like lipid composition, PR AUC is preferable (Saito & Rehmsmeier, 2015). Decision tree (0.936), nearest neighbor (0.935), trimmed decision tree (0.929), and random forest (0.920) show the highest PR AUC for the outcome “non-lipid”. The PR AUC for “lipid” shows the lowest performance among the models evaluated, with the best results coming from the SVM model (0.651), followed closely by boosted trees (0.649), naïve Bayes (0.646), and ridge regression (0.644) (see Fig. 1E and Table 1).

**Table 1:** Ranked performance of 10 models on the testing set based on accuracy, ROC AUC, PR AUC “non-lipid”, PR AUC “lipid”

Models	Final rank	Rank sum	Accuracy	Accuracy rank	ROC AUC	ROC AUC rank	PR AUC (No)	PR AUC (No) rank	PR AUC (Yes)	PR AUC (Yes) rank
Decision Tree	1	14.5	0.865	1.5	0.771	3	0.936	1	0.511	9
Trimmed Decision Tree	2	18.5	0.865	1.5	0.727	4	0.929	3	0.443	10
Random Forest	3	19	0.838	6	0.782	2	0.92	4	0.619	7
KNN	4	20	0.811	9	0.809	1	0.935	2	0.595	8
Elasticnet	5.5	23	0.838	6	0.692	5	0.849	6.5	0.643	5.5
Ridge regression	5.5	23	0.851	3.5	0.69	7	0.848	8.5	0.644	4
Lasso regression	7	24	0.838	6	0.691	6	0.849	6.5	0.643	5.5
SVM	8	24.5	0.851	3.5	0.672	10	0.837	10	0.651	1
Naive Bayes	9	26	0.797	10	0.686	8	0.863	5	0.646	3
Boosted Trees	10	27.5	0.824	8	0.673	9	0.848	8.5	0.649	2

Considering the overall performance ranking based on the 4 metrics, the best models were decision tree, trimmed decision tree, random forest, and nearest neighbor (Table 1). Manual comparison of the performance on the training set and the testing set indicated that, as expected, most models show more accurate predictions on the training data compared with the testing data, while the trimmed decision tree performed similarly on the training and testing dataset (Fig. 1E and Table S4).

### Models’ validation and prediction

**Fig. 2:**
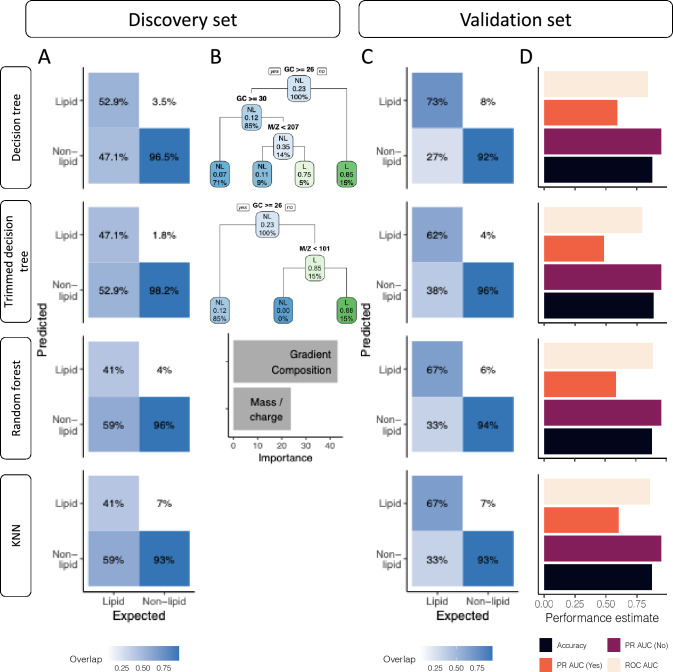
Predictions of the 4 best models on the discovery and validation sets. **A**. Confusion matrices showing model predictions on the discovery set. **B**. Variable importance estimated on the discovery set (abbreviations in trees; NL: “Non-lipid”, L: “Lipid”). Variable importance for KNN models cannot be assessed. **C**. Confusion matrices showing model predictions on the validation set. **D**. Performance on the validation set including accuracy, ROC AUC, PR AUC (“non-lipid”, “lipid”) metrics

For the top 4 models, based on the confusion matrix, predictions on the discovery data revealed high accuracy for the “non-lipid” outcome, ranging from 93% (KNN, 53/57) to 98.2% (trimmed decision tree, 56/57) (Fig. 2A). Conversely, predictions for the “lipid” outcome were lower, with the decision tree achieving the highest accuracy at 52.9% (9/17) followed by trimmed decision tree (47.1%, 8/17), random forest (41%, 7/17), and KNN (41%, 7/17) (Fig. 2A). The tree chart and variable importance visualization show the importance of the gradient composition predictor over the *m/z* predictor for assigning lipids (Fig. 2B). For the random forest model, the gradient composition variable was almost twice as important as the *m/z* variable. In both decision tree models, the gradient composition variable served as the first node, splitting the root node into a “lipid” branch for a value < 26 and a “non-lipid” branch for a value ≥ 26 (Fig. 2B). Similar prediction patterns were observed based on the confusion matrix in the validation data, with accurate predictions for the “non-lipid” outcome (92–96%, 413/450- 432/450) and lower predictions for the “lipid” outcome (62–73%, 82/132–97/132; Fig. 2C). Notably, the trimmed decision tree displayed the highest accurate prediction for the “non-lipid” outcome, but the lowest for the “lipid” outcome (96% [432/450] and 62% [82/132] respectively, Fig. 2C). Inversely, the decision tree model achieved the highest value for the “lipid” outcome but the lowest prediction for the “non-lipid” outcome (73% [97/132] and 92% [413/450] respectively, Fig. 2C). A manual visual comparison of correctly and incorrectly classified metabolites shows that “non-lipid” metabolites with lower gradient concentration are incorrectly classified while metabolites with low gradient concentration are often correctly classified as “lipid”. The inverse was observed for metabolites with higher gradient concentrations (Figure S4).

Performance on the validation set is similar to the performance observed in the training set. Across all models, there was a trend of higher performance for the PR AUC (“non-lipid”) metric and lower for the PR AUC (“lipid”) metric (Fig. 2D). The trimmed decision tree achieved the highest accuracy and exhibited the lowest PR AUC (“lipid”). Interestingly, the nearest neighbor model shows the highest PR AUC (“lipid”) performance (0.602), followed by decision tree (0.591), random forest (0.589), and trimmed decision tree (0.482) (Fig. 2D and Table 2).

**Table 2:** Performance of the 4 best models on the validation set

Performance Metrics	Random Forest	Decision Tree	Trimmed Decision Tree	KNN
Accuracy	0.881	0.876	0.883	0.873
PR AUC (No)	0.958	0.947	0.944	0.946
PR AUC (Yes)	0.589	0.591	0.482	0.602
ROC AUC	0.891	0.84	0.791	0.854

We established a machine learning framework that is broadly applicable to unidentified metabolite classification in untargeted metabolomics. This framework can easily be extended to classify a wide range of metabolites using only *m/z* and RT. We show that this approach is independent of the chromatography method. To ensure consistency, both datasets underwent the same cleaning processes to reduce false detections. We tested a combination of four data preprocessing techniques, a no-preprocessing approach, and 10 machine learning models on a discovery and two validation datasets. Performance evaluation ranking based on accuracy, ROC AUC, and both PR AUC for each outcome (“lipid” and “non-lipid”) pointed to the best models being decision tree, trimmed decision tree, random forest, and nearest neighbor. Across these models, the trimmed decision tree showed a similar performance in the training and testing sets which indicates that the model was not overfitting. In contrast, this model exhibited the least accurate prediction for the “lipid” outcome. The decision tree model achieved the most accurate prediction for the “lipid” outcome; however, its performance indicated mild overfitting, primarily when assessed by ROC AUC. Performance discrepancies between the discovery and validation datasets may be driven by the availability of fewer samples during the training process which may result in overfitting on the discovery dataset. In general, tree-based models, often perform well on tabular data due to their ability to capture non-linear relationships and handle feature interactions without requiring normalization. In our study, simpler tree models outperformed others likely due to the small dataset size and limited number of features (m/z and RT), where complex ensemble methods may overfit or offer limited benefit over simpler models.

We next applied the framework to an independent dataset of human plasma samples from healthy women, collected under distinct experimental conditions, to demonstrate its robustness across studies. This new dataset exhibited marked differences in RT distribution compared to the original training and validation datasets. Despite these variations, the framework was successfully extended to the new dataset, underscoring its adaptability. Random forest, boosted trees and Naive Bayes performed best on accuracy (0.949) followed by SVM (0.915), KNN (0.915), elastic net (0.881), lasso regression (0.881), trimmed and un-trimmed Decision tree (0.881), and ridge regression (0.864). A similar ranking is observed for ROC AUC and PR AUC “non-lipid”, with the best models being random forest (0.986 and 0.993, respectively), boosted trees (0.977 and 0.988, respectively) and Naive Bayes (0.975 and 0.986, respectively) and SVM (0.962 and 0.977, respectively). The performance for PR AUC “lipid” ranked elastic net and lasso regression (0.443) the highest followed by ridge regression (0.441), and SVM (0.435) (Figure S6A-D and Table S5). Between the top 4 models, confusion matrix results show that boosted trees exhibit the highest predictions accuracy for the “lipid” outcome (95.24%, 20/21) while random forest and naïve bayes predict best the “non-lipid” outcome (97.4%, 37/38) (Figure S6A-D).

As expected, limited retraining or fine-tuning was required to maintain predictive performance. These findings support our hypothesis that while complete model portability across LC–MS platforms and acquisition methods may necessitate limited retraining, the underlying framework remains robust and broadly applicable. In this dataset, we also observed slight overfitting with Naïve Bayes and the Trimmed Decision Tree, the latter showing the lowest prediction accuracy for the “lipid” class, consistent with our mouse sample results (Figure S5). Additionally, preprocessing steps such as dimensionality reduction, normalization, scaling, or their combinations did not improve performance, in agreement with observations from our mouse data (Figure S6A-D).

Future work will focus on testing this framework on other classification tasks as such as more detailed taxonomy class predictions and metabolic pathways. Furthermore, given the established link between lipid metabolism and cancer prognosis (Santos & Schulze, 2012), as well as its potential as a target for antineoplastic therapies, a future direction of this work is to explore the association between lipid prediction outcomes and cancer prognosis. This investigation could provide valuable insights to support decision-making in metabolism-targeted treatments.

In conclusion, we lay the groundwork in the prediction of metabolites superclass classification of unknown metabolites using minimal data. We developed a robust framework based on a broad comparison of diverse preprocessors in combination with several machine learning models. Moreover, we evaluated them on the best metrics for our specific use case. These steps are essential for narrowing down to the best algorithm that suits the data and the application. We show that this approach is independent of the chromatography and future work will establish if this framework will perform well with other classification tasks.

## Supplementary Information

Below is the link to the electronic supplementary material.

Supplementary file1 (PPTX 51 KB)

Supplementary file2 (PPTX 262 KB)

Supplementary file3 (PDF 7 KB)

Supplementary file4 (PPTX 248 KB)

Supplementary file5 (PPTX 105 KB)

Supplementary file6 (PPTX 263 KB)

Supplementary file7 (XLSX 61 KB)

Supplementary file8 (XLSX 17 KB)

Supplementary file9 (XLSX 11 KB)

Supplementary file10 (XLSX 11 KB)

Supplementary file11 (XLSX 10 KB)

## Data Availability

Data from this study was deposited to Zenodo with 10.5281/zenodo.17227184.

## References

[CR1] Almutairi, M. H., Alrubie, T. M., Alshareeda, A. T., Albarakati, N., Almotiri, A., Alamri, A. M., Almutairi, B. O., & Alanazi, M. (2024). Differential expression and regulation of ADAD1, DMRTC2, PRSS54, SYCE1, SYCP1, TEX101, TEX48, and TMPRSS12 gene profiles in colon cancer tissues and their in vitro response to epigenetic drugs. *PLoS ONE,**19*(8), e0307724. 39208330 10.1371/journal.pone.0307724 PMC11361649

[CR2] Bowen, B. P., & Northen, T. R. (2011). Dealing with the unknown: Metabolomics and metabolite atlases. *Journal of the American Society for Mass Spectrometry*. 10.1016/j.jasms.2010.04.003 10.1016/j.jasms.2010.04.003 20452782

[CR3] de Cripan, S. M., Arora, T., Olomí, A., Canela, N., Siuzdak, G., & Domingo-Almenara, X. (2024). Predicting the predicted: A comparison of machine learning-based collision cross-section prediction models for small molecules. *Analytical Chemistry,**96*(22), 9088–9096. 38783786 10.1021/acs.analchem.4c00630 PMC11154685

[CR4] Dührkop, K., Fleischauer, M., Ludwig, M., Aksenov, A. A., Melnik, A. V., Meusel, M., Dorrestein, P. C., Rousu, J., & Böcker, S. (2019). SIRIUS 4: a rapid tool for turning tandem mass spectra into metabolite structure information. *Nature Methods,**16*(4), 299–302. 30886413 10.1038/s41592-019-0344-8

[CR5] Dührkop, K., Shen, H., Meusel, M., Rousu, J., & Böcker, S. (2015). Searching molecular structure databases with tandem mass spectra using CSI:FingerID. *Proceedings of the National Academy of Sciences U S A,**112*(41), 12580–12585. 10.1073/pnas.1509788112 PMC4611636 26392543

[CR6] Galal, A., Talal, M., & Moustafa, A. (2022). Applications of machine learning in metabolomics: Disease modeling and classification. *Frontiers in Genetics,**13*, 1017340. 36506316 10.3389/fgene.2022.1017340 PMC9730048

[CR7] Kaczmarek, M., Zhang, N., Buzhansky, L., Gilead, S., & Gazit, E. (2023). Optimization strategies for mass spectrometry-based untargeted metabolomics analysis of small polar molecules in human plasma. *Metabolites,**13*(8), 923. 37623867 10.3390/metabo13080923 PMC10456887

[CR8] Kuhn, M. and H. Wickham, *Tidymodels: a collection of packages for modeling and machine learning using tidyverse principles*. 2020

[CR9] Li, S., Park, Y., Duraisingham, S., Strobel, F. H., Khan, N., Soltow, Q. A., Jones, D. P., & Pulendran, B. (2013). Predicting network activity from high throughput metabolomics. *PLoS Computational Biology,**9*(7), e1003123. 23861661 10.1371/journal.pcbi.1003123 PMC3701697

[CR10] Ma, G., Kang, J., & Yu, T. (2024). Bayesian functional analysis for untargeted metabolomics data with matching uncertainty and small sample sizes. *Brief Bioinformatics*. 10.1093/bib/bbae141 38581417 10.1093/bib/bbae141 PMC10998539

[CR11] Mascanfroni, I. D., Takenaka, M. C., Yeste, A., Patel, B., Wu, Y., Kenison, J. E., Siddiqui, S., Basso, A. S., Otterbein, L. E., Pardoll, D. M., & Pan, F. (2015). Metabolic control of type 1 regulatory T cell differentiation by AHR and HIF1-alpha. *Nature Medicine,**21*(6), 638–646. 26005855 10.1038/nm.3868 PMC4476246

[CR12] O’Sullivan, J. F., Morningstar, J. E., Yang, Q., Zheng, B., Gao, Y., Jeanfavre, S., Scott, J., Fernandez, C., Zheng, H., O’Connor, S., & Cohen, P. (2017). Dimethylguanidino valeric acid is a marker of liver fat and predicts diabetes. *Journal of Clinical Investigation,**127*(12), 4394–4402. 29083323 10.1172/JCI95995 PMC5707166

[CR13] Paynter, N. P., Balasubramanian, R., Giulianini, F., Wang, D. D., Tinker, L. F., Gopal, S., Deik, A. A., Bullock, K., Pierce, K. A., Scott, J., & Martínez-González, M. A. (2018). Metabolic predictors of incident coronary heart disease in women. *Circulation,**137*(8), 841–853. 29459470 10.1161/CIRCULATIONAHA.117.029468 PMC5854187

[CR14] Pirhaji, L., Milani, P., Leidl, M., Curran, T., Avila-Pacheco, J., Clish, C. B., White, F. M., Saghatelian, A., & Fraenkel, E. (2016). Revealing disease-associated pathways by network integration of untargeted metabolomics. *Nature Methods,**13*(9), 770–776. 27479327 10.1038/nmeth.3940 PMC5209295

[CR15] Pluskal, T., Castillo, S., Villar-Briones, A., & Orešič, M. (2010). MZmine 2: modular framework for processing, visualizing, and analyzing mass spectrometry-based molecular profile data. *BMC Bioinformatics,**11*, 395. 20650010 10.1186/1471-2105-11-395 PMC2918584

[CR16] Rivas-Ubach, A., Liu, Y., Bianchi, T. S., Tolic, N., Jansson, C., & Pasa-Tolic, L. (2018). Moving beyond the van Krevelen Diagram: A new stoichiometric approach for compound classification in organisms. *Analytical Chemistry,**90*(10), 6152–6160. 29671593 10.1021/acs.analchem.8b00529

[CR17] Saito, T., & Rehmsmeier, M. (2015). The precision-recall plot is more informative than the ROC plot when evaluating binary classifiers on imbalanced datasets. *PLoS ONE*. 10.1371/journal.pone.0118432 25738806 10.1371/journal.pone.0118432 PMC4349800

[CR18] Santos, C. R., & Schulze, A. (2012). Lipid metabolism in cancer. *FEBS Journal,**279*(15), 2610–2623. 22621751 10.1111/j.1742-4658.2012.08644.x

[CR19] Townsend, M. K., Clish, C. B., Kraft, P., Wu, C., Souza, A. L., Deik, A. A., Tworoger, S. S., & Wolpin, B. M. (2013). Reproducibility of metabolomic profiles among men and women in 2 large cohort studies. *Clinical Chemistry,**59*(11), 1657–1667. 23897902 10.1373/clinchem.2012.199133 PMC3812240

[CR20] Welsh, E. A., Eschrich, S. A., Berglund, A. E., & Fenstermacher, D. A. (2013). Iterative rank-order normalization of gene expression microarray data. *BMC Bioinformatics,**14*, 153. 23647742 10.1186/1471-2105-14-153 PMC3651355

[CR21] Wishart, D. S., Guo, A., Oler, E., Wang, F., Anjum, A., Peters, H., Dizon, R., Sayeeda, Z., Tian, S., Lee, B. L., & Berjanskii, M. (2022). HMDB 5.0: The human metabolome database for 2022. *Nucleic Acids Research,**50*(D1), D622–D631. 34986597 10.1093/nar/gkab1062 PMC8728138

[CR22] Zeleznik, O. A., Eliassen, A. H., Kraft, P., Poole, E. M., Rosner, B. A., Jeanfavre, S., Deik, A. A., Bullock, K., Hitchcock, D. S., Avila-Pacheco, J., & Clish, C. B. (2020). A prospective analysis of circulating plasma metabolites associated with ovarian cancer risk. *Cancer Research,**80*(6), 1357–1367. 31969373 10.1158/0008-5472.CAN-19-2567 PMC7073287

