# Supplementary material for: A machine learning framework for classifying lipids in untargeted metabolomics using mass-to-charge ratios and retention times
Source: Metabolomics. 2025 Oct 18;21(6):151. doi: 10.1007/s11306-025-02343-y (PMC12535499; doi:10.1007/s11306-025-02343-y)
Supplement: Supplementary file 1 — Supplementary file1 (PPTX 51 KB) [file 11306_2025_2343_MOESM1_ESM.pptx]

## Slide 1
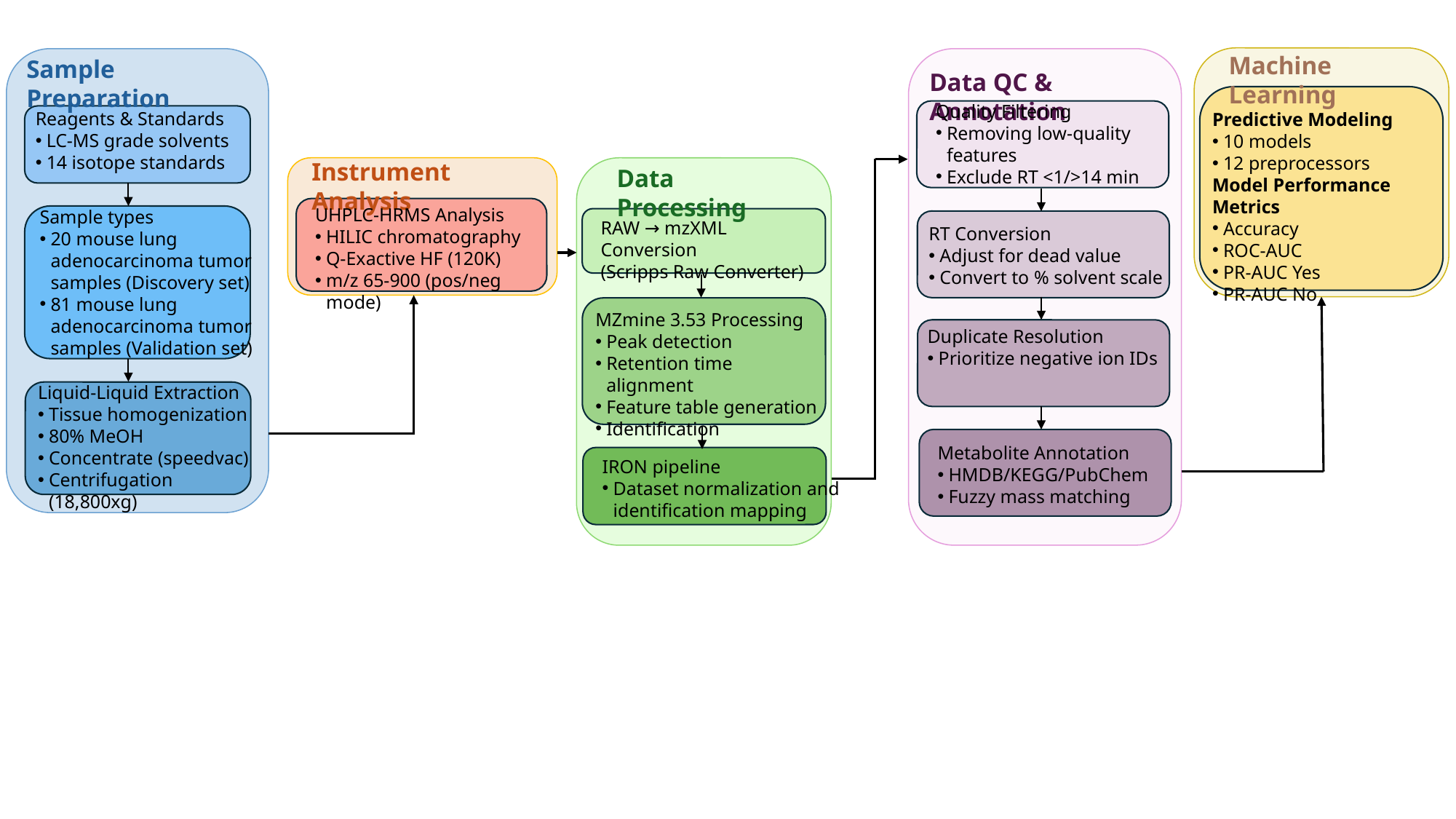

Machine Learning
Sample Preparation
Data QC & Annotation
Quality Filtering
Removing low-quality features
Exclude RT <1/>14 min
Reagents & Standards
LC-MS grade solvents
14 isotope standards
Predictive Modeling
10 models
12 preprocessors
Model Performance Metrics
Accuracy
ROC-AUC
PR-AUC Yes
PR-AUC No
Instrument Analysis
Data Processing
UHPLC-HRMS Analysis
HILIC chromatography
Q-Exactive HF (120K)
m/z 65-900 (pos/neg mode)
Sample types
20 mouse lung adenocarcinoma tumor samples (Discovery set)
81 mouse lung adenocarcinoma tumor samples (Validation set)
RAW → mzXML Conversion
(Scripps Raw Converter)
RT Conversion
Adjust for dead value
Convert to % solvent scale
MZmine 3.53 Processing
Peak detection
Retention time alignment
Feature table generation
Identification
Duplicate Resolution
Prioritize negative ion IDs
Liquid-Liquid Extraction
Tissue homogenization
80% MeOH
Concentrate (speedvac)
Centrifugation (18,800xg)
Metabolite Annotation
HMDB/KEGG/PubChem
Fuzzy mass matching
IRON pipeline
Dataset normalization and identification mapping
