# Supplementary figures and images for: A machine learning framework for classifying lipids in untargeted metabolomics using mass-to-charge ratios and retention times
Source: Metabolomics. 2025 Oct 18;21(6):151. doi: 10.1007/s11306-025-02343-y (PMC12535499; doi:10.1007/s11306-025-02343-y)

## Slide 1
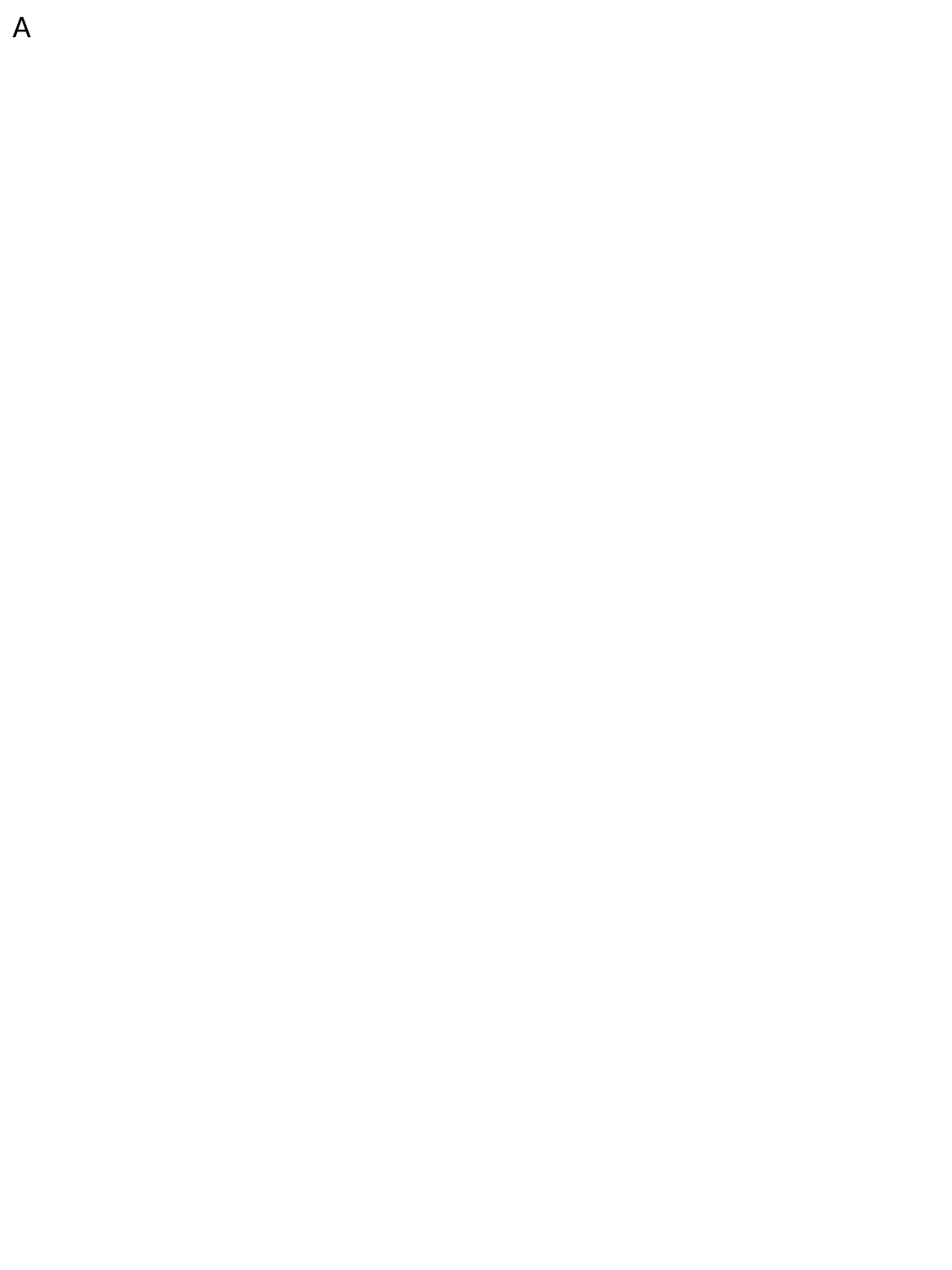

A

## Slide 2
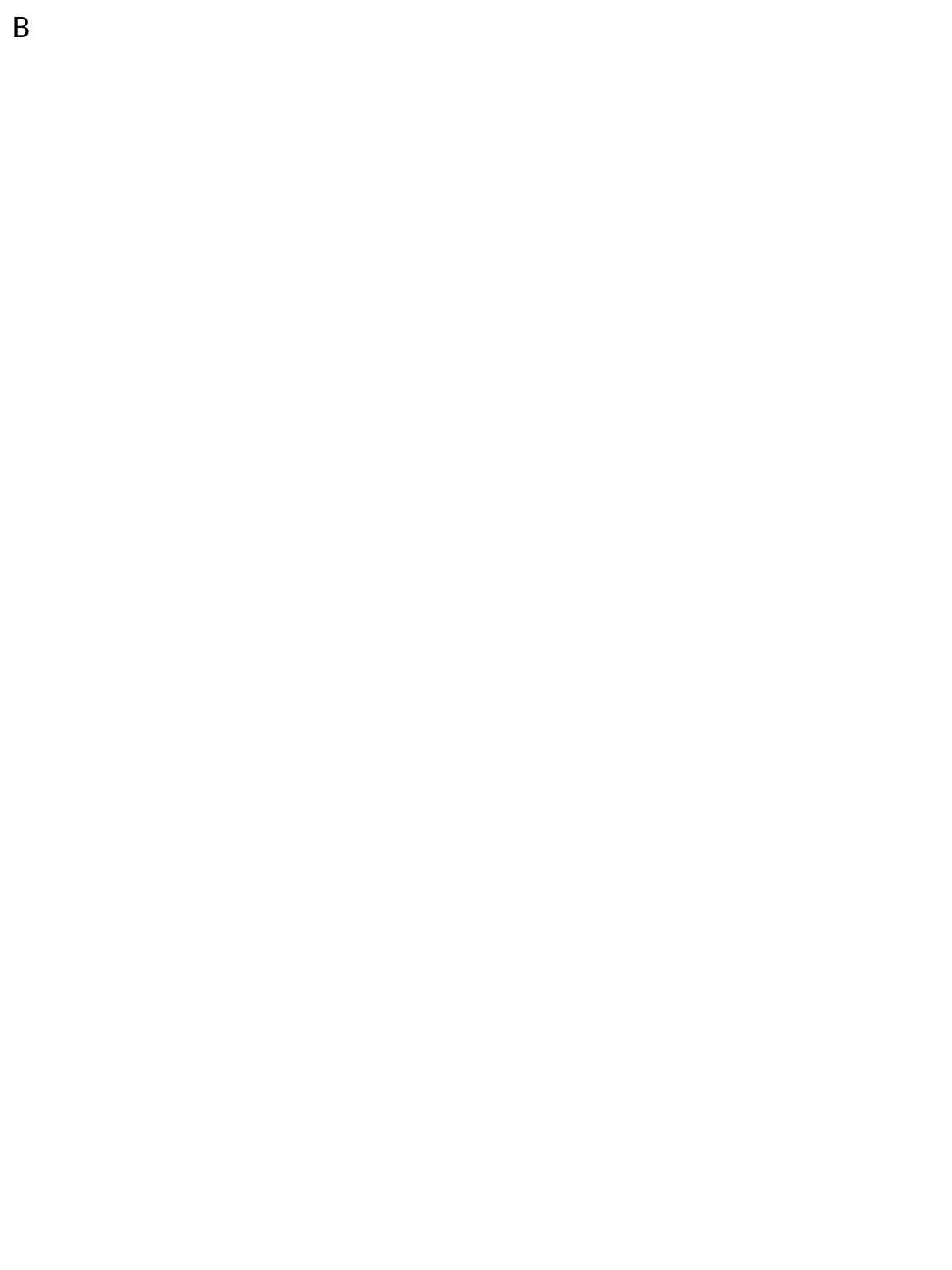

B

## Slide 3
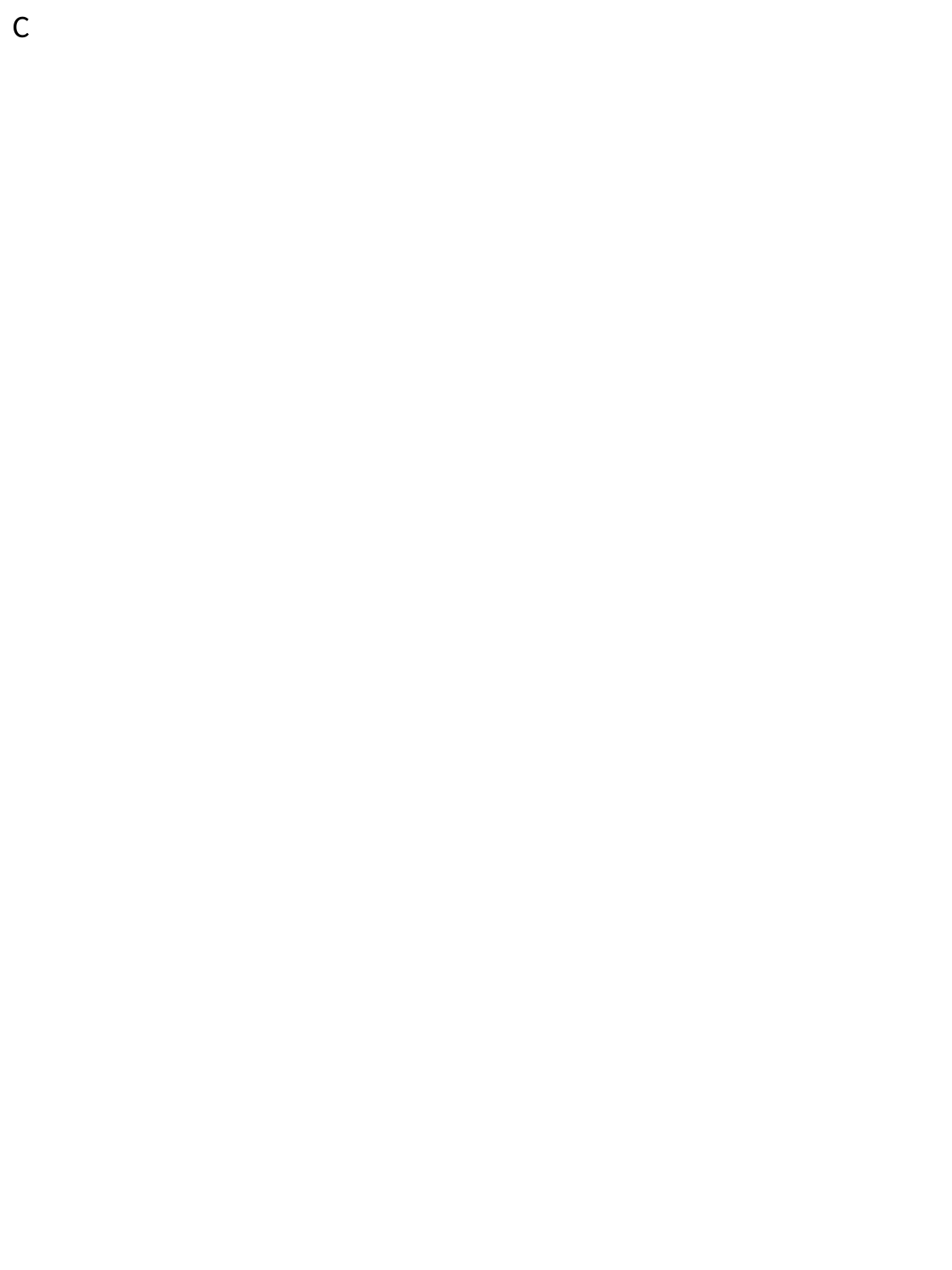

C

## Slide 4
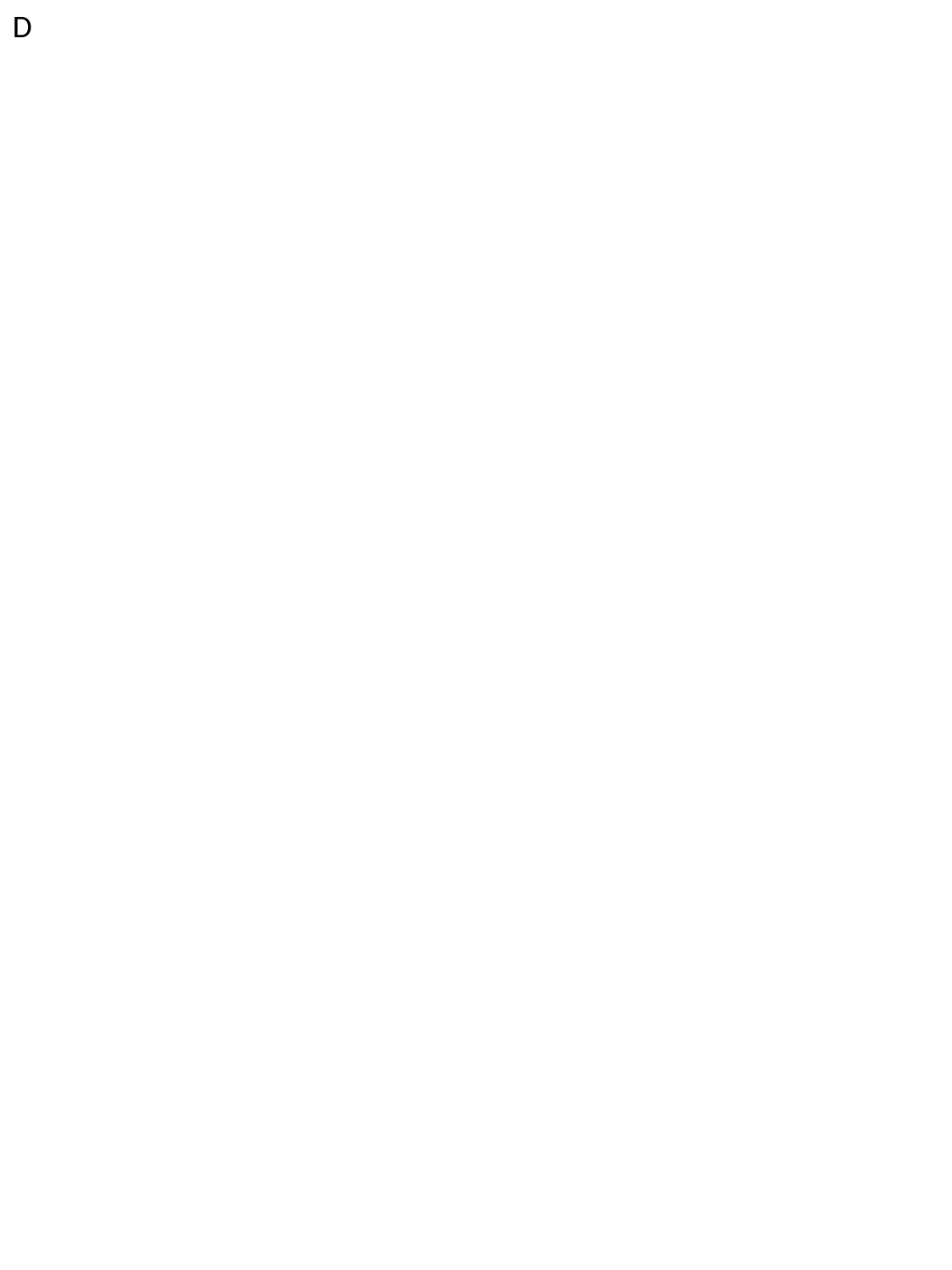

D

Supplement: Supplementary file 2 — Supplementary file2 (PPTX 262 KB) [file 11306_2025_2343_MOESM2_ESM.pptx]

A

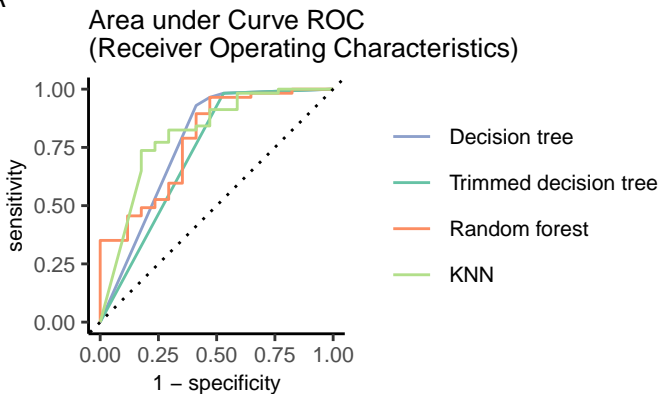

B

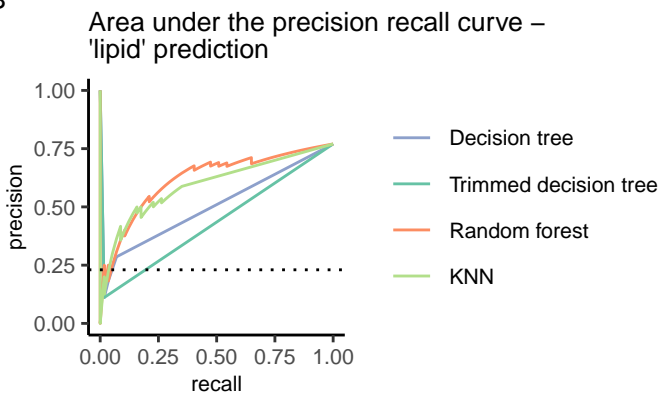

C

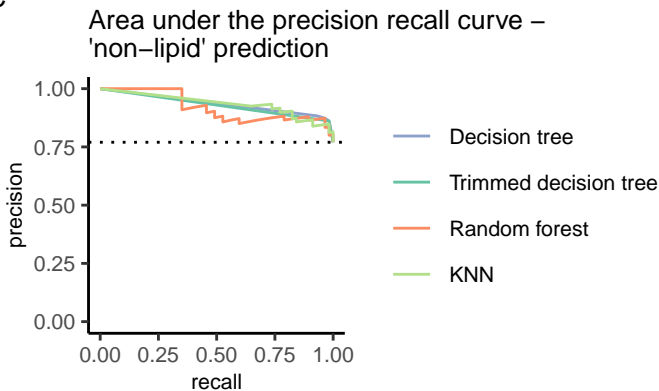

Supplement: Supplementary file 3 — Supplementary file3 (PDF 7 KB) [file 11306_2025_2343_MOESM3_ESM.pdf]

## Slide 1
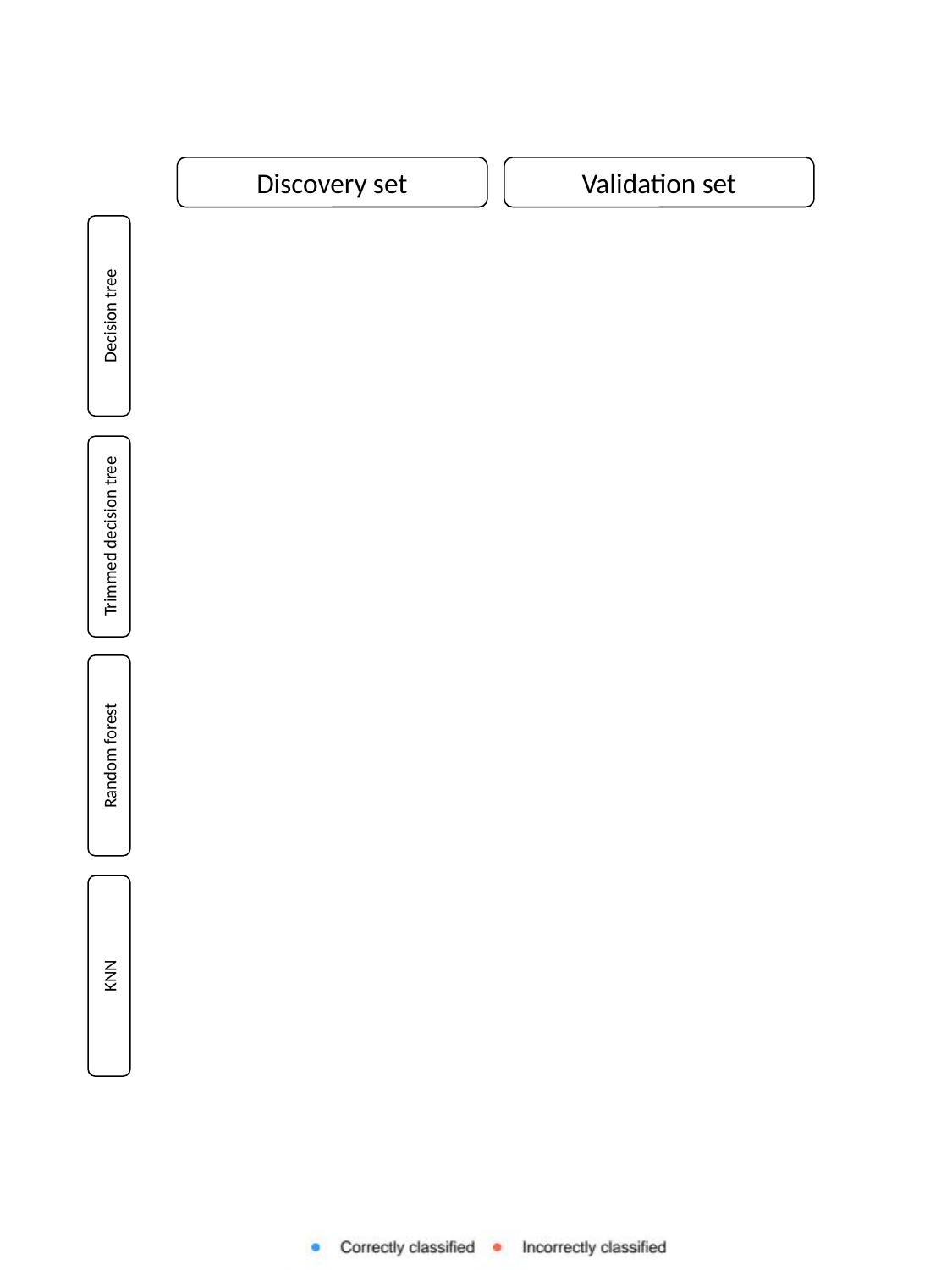

Discovery set
Validation set
Decision tree
Trimmed decision tree
Random forest
KNN

Supplement: Supplementary file 4 — Supplementary file4 (PPTX 248 KB) [file 11306_2025_2343_MOESM4_ESM.pptx]

## Slide 1
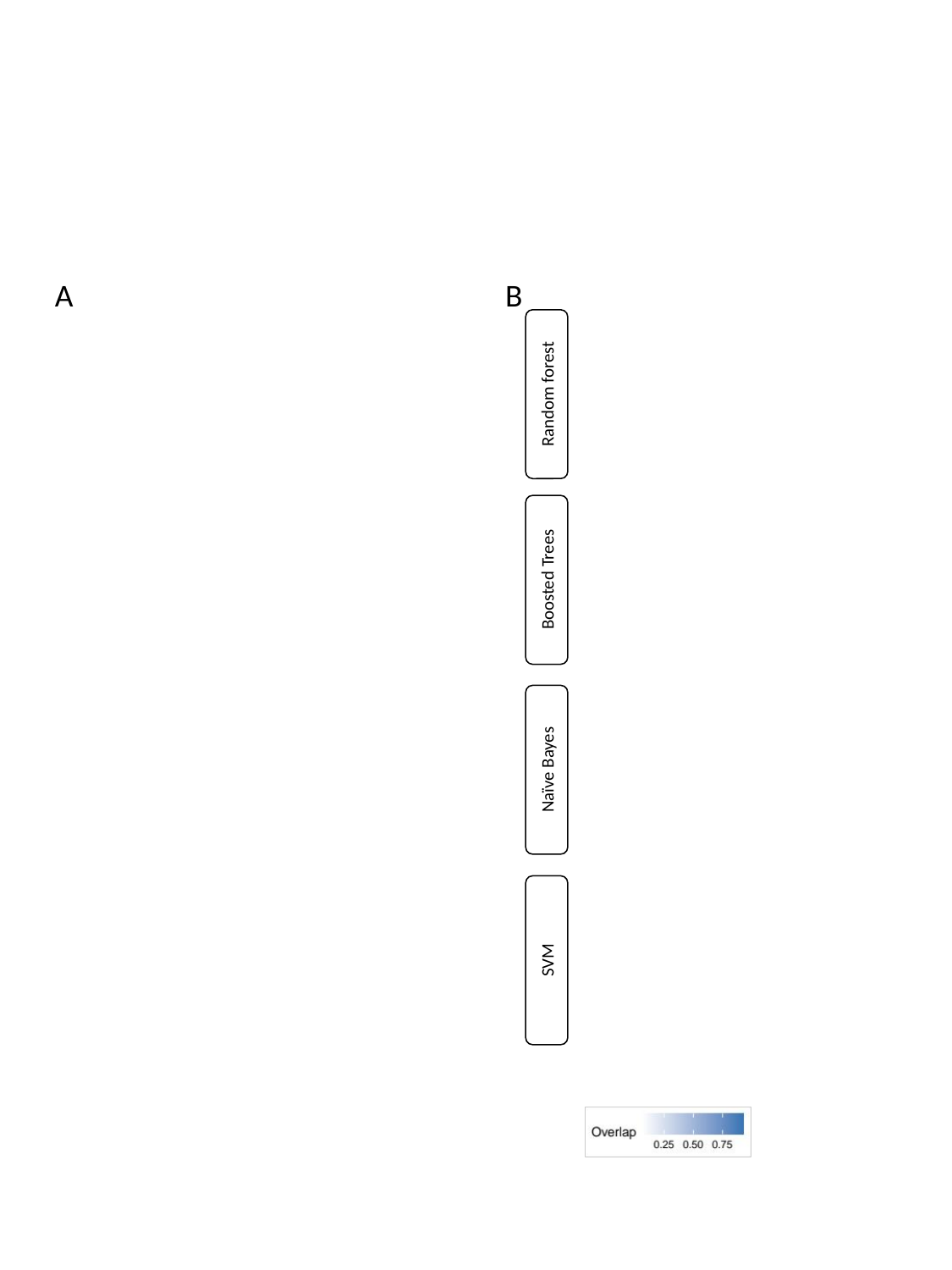

A
B
Random forest
Boosted Trees
Naïve Bayes
SVM

Supplement: Supplementary file 5 — Supplementary file5 (PPTX 105 KB) [file 11306_2025_2343_MOESM5_ESM.pptx]

## Slide 1
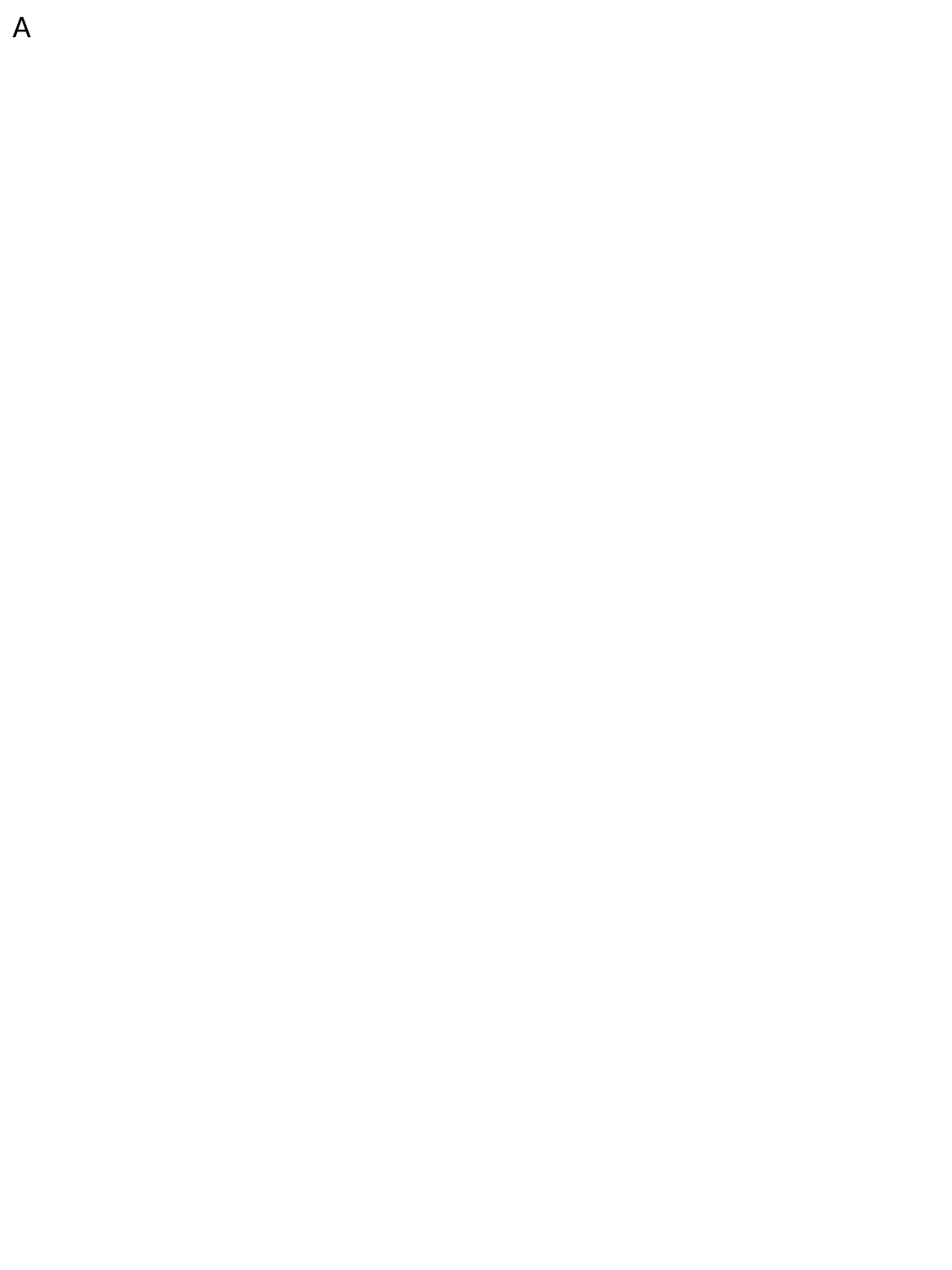

A

## Slide 2
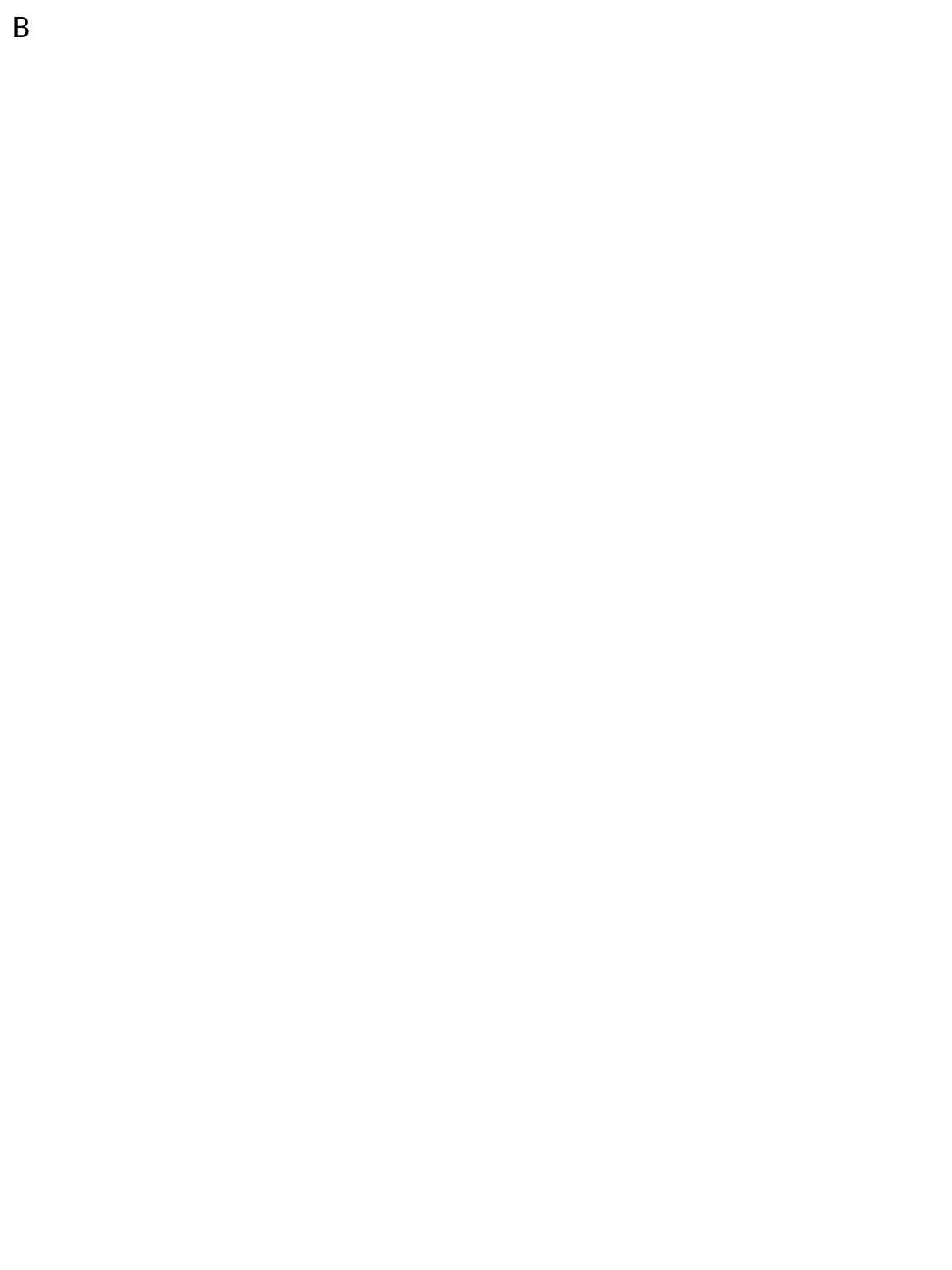

B

## Slide 3
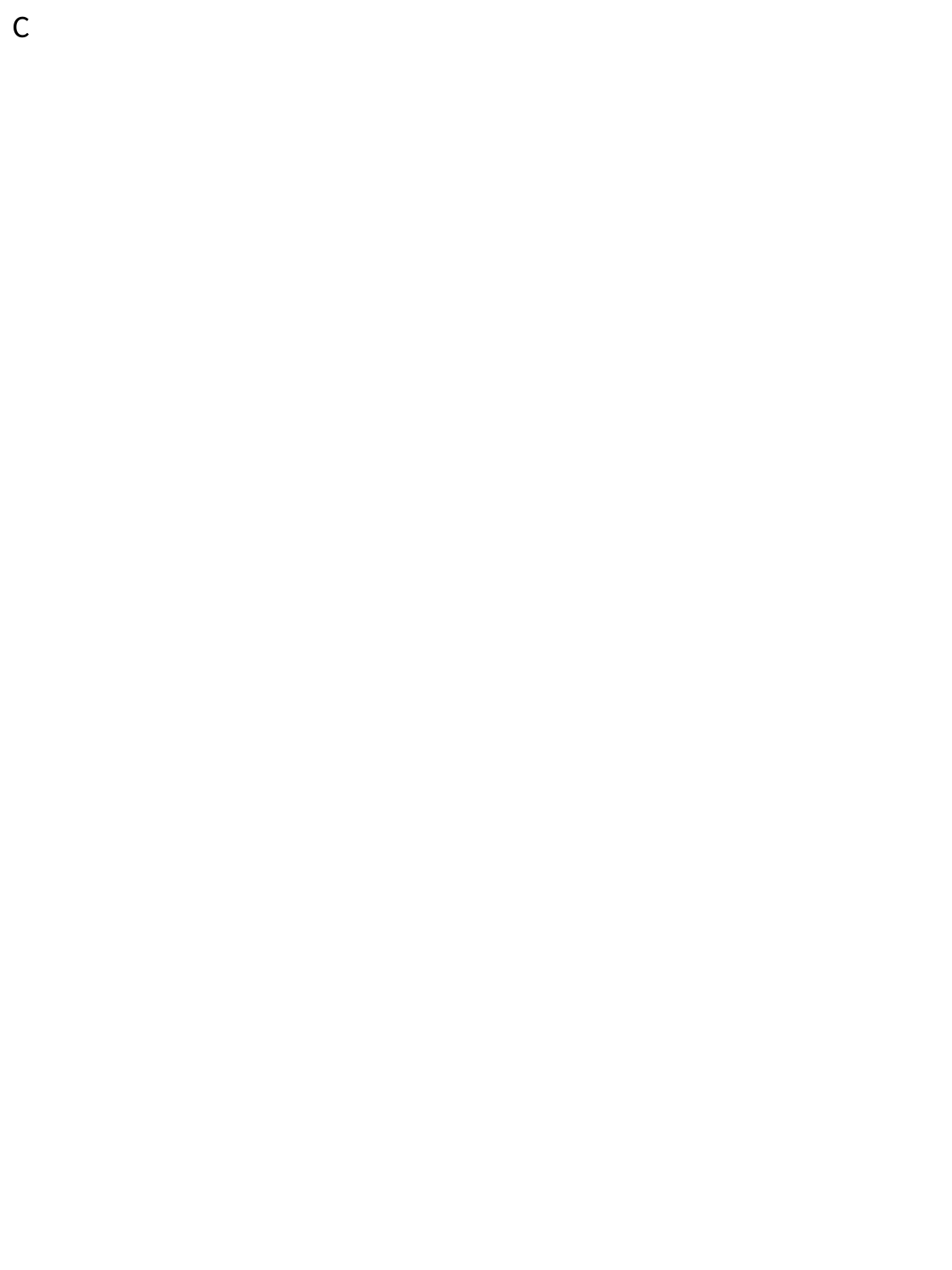

C

## Slide 4
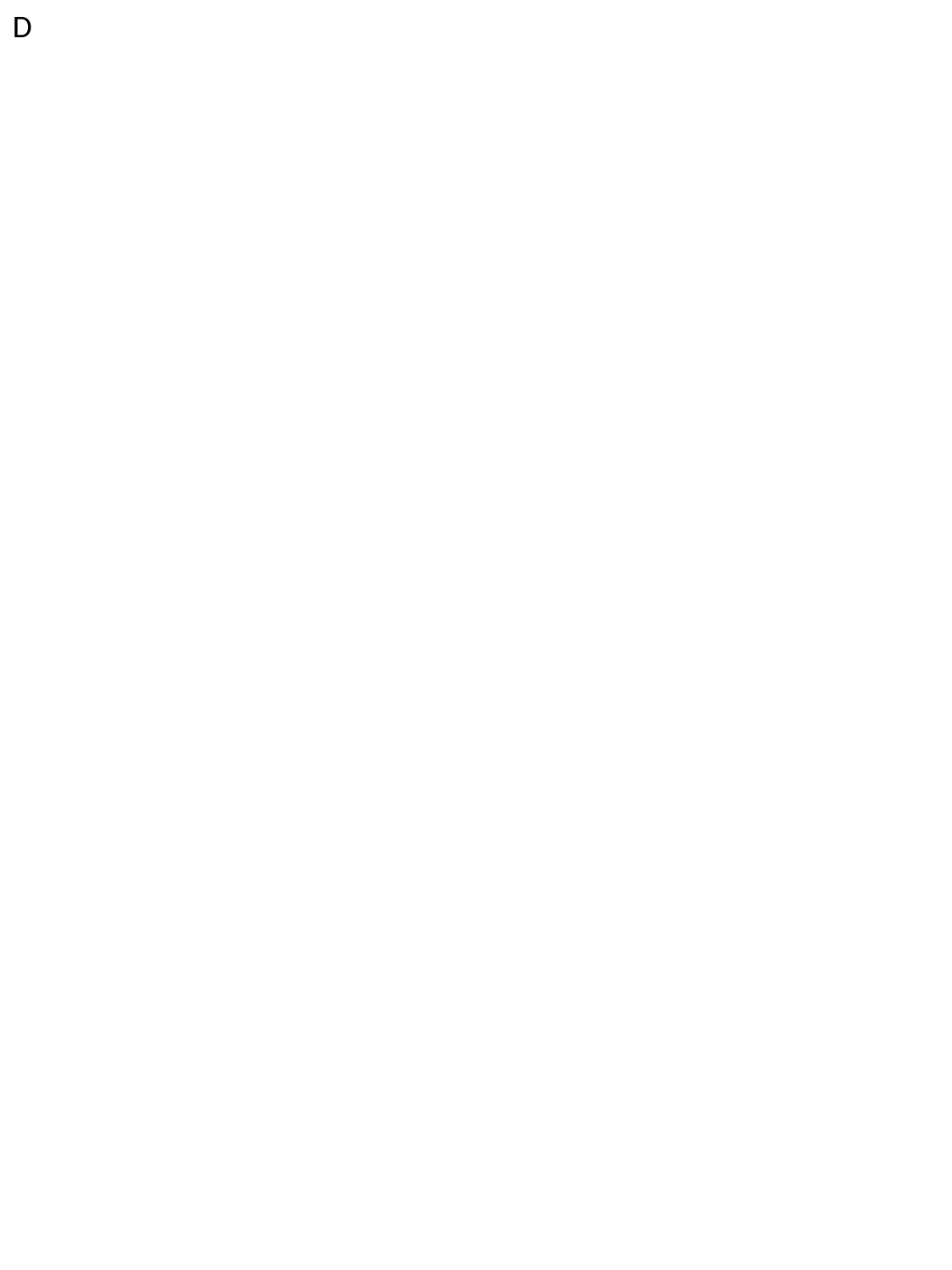

D

Supplement: Supplementary file 6 — Supplementary file6 (PPTX 263 KB) [file 11306_2025_2343_MOESM6_ESM.pptx]
